# Instrument for assessing social network support for women with breast cancer

**DOI:** 10.1590/0034-7167-2025-0148

**Published:** 2026-07-27

**Authors:** Diego Augusto Lopes Oliveira, Vânia Pinheiro Ramos, Luciana Pedrosa Leal, Gabriela Cunha Schechtman Sette, Wilson Jorge Correia Pinto de Abreu, Cleide Maria Pontes

**Affiliations:** I Universidade de Pernambuco. Recife, Pernambuco, Brazil; II Universidade Federal de Pernambuco. Recife, Pernambuco, Brazil; III Escola Superior de enfermagem do Porto. Porto, Portugal

**Keywords:** Breast Neoplasms, Validity Study, Drug Therapy, Social Networking, Social Support., Neoplasias de la Mama, Estudio de Validación, Quimioterapia, Red Social, Apoyo Social.

## Abstract

**Objectives:**

to validate an instrument for assessing social network support for women with breast cancer undergoing chemotherapy.

**Methods:**

a methodological study with construction stages based on Social Network Theory. Content validity was performed by ten judges (nominal group), and semantic assessment was conducted by eight women (brainstorming). Data were analyzed using relative frequencies, Content Validity Coefficient, Intraclass Correlation Coefficient, and Confidence Index.

**Results:**

the instrument, with five dimensions of support (emotional/esteem, informational, tangible/instrumental, companionship, and self-support), had 60 items. Most items had coefficients ≥0.80 in judges’ assessment. After eliminating items and making adjustments, the instrument was left with 47 items, with a semantic assessment that reached a Confidence Index of 0.99. Adjustments were made for greater clarity, maintaining the 47 items across the five support dimensions.

**Conclusions:**

the instrument was validated as a tool to assess social network support for women with breast cancer undergoing chemotherapy.

## RESUMEN

**Objetivos:**

validar un instrumento para evaluar el apoyo de las redes sociales en mujeres con cáncer de mama en quimioterapia.

**Métodos:**

estudio metodológico con etapas de construcción basadas en la Teoría de Redes Sociales. La validez de contenido fue realizada por diez jueces (grupo nominal) y la evaluación semántica por ocho mujeres (*brainstorming*). Los datos se analizaron mediante frecuencias relativas, coeficiente de validez de contenido, coeficiente de correlación intraclase e índice de confianza.

**Resultados:**

el instrumento, con cinco dimensiones de apoyo (emocional, informativo, instrumental, presencial y autoapoyo), contaba con 60 ítems. La mayoría presentó coeficientes ≥0,80 en la evaluación de los jueces. Tras la eliminación de ítems y los ajustes, el instrumento quedó con 47 ítems, con una evaluación semántica que alcanzó un Índice de Confianza de 0,99. Se realizaron ajustes para mayor claridad, manteniendo los 47 ítems en las cinco dimensiones de apoyo.

**Conclusiones:**

el instrumento se validó como herramienta para evaluar el apoyo de las redes sociales en mujeres con cáncer de mama en quimioterapia.

## INTRODUCTION

Breast cancer is the neoplasm that causes the most deaths among women worldwide, with approximately 2.3 million deaths reported in 2020^(1)^. The increase in the number of cases is linked to population aging, changes in consumption patterns, lifestyle, and weak public policies related to early diagnosis and screening of the disease^(2)^. This situation hinders the development of a care pathway that would allow for better treatment and prognosis for affected women^(1,2)^.

The established strategies for approaching and intervening in breast cancer management highlight chemotherapy as a treatment that optimizes time and delays tumor progression. Its use involves the administration of drugs that produce toxic effects on cellular DNA. Its use also brings consequences for women, leading to adverse reactions that imply changes in their physical and mental well-being, quality of life, and social interaction, especially in maintaining relationships and receiving support from their social network^(3,4)^.

A person’s social network is formed from the connections established in their daily life, shaped by the relationship ties that promote partnership, exchange, and social support sharing. The support offered by the social network in the emotional/esteem (empathetic, caring, and concerned aspects of women), companionship (making time available to accompany women, providing support, and fostering a sense of belonging to a social group), informational (advice, guidance, suggestions, and feedback on women’s behavior in facing the challenges imposed by illness), tangible/instrumental (practical help to replace daily activities that cannot be performed due to illness), and self-support dimensions (individual decisions made by women and motivation to cope with treatments, the challenges of the disease, and adherence to proposals for improving their health) strengthens women’s ability to cope with cancer and chemotherapy^(5-7)^.

The social relationships of a woman diagnosed with breast cancer undergo significant transformations throughout chemotherapy, sometimes weakening her social bonds, and other times strengthening them. This movement is directly related to the support provided by the social network, so its assessment is necessary in order to demand measures that strengthen support practices and culminate in benefits in the care process throughout the use of antineoplastic drugs^(8)^.

Assessing the social support network for women with breast cancer during chemotherapy is a strategy that contributes to comprehensive care through non-procedural actions involving family, friends, neighbors, and healthcare professionals in promoting well-being, quality of life, and overcoming difficult situations experienced during the journey of facing a neoplasm^(5)^.

Care technology construction and validity, especially assessment instruments, have been increasingly used as a way to present scientifically robust results regarding care actions for individuals, families, and communities. The importance of adhering to theoretical and methodological rigor is highlighted in order to achieve an appropriate instrument that aims to measure/assess and that has criteria for constructing assessment items^(9)^. Given this context, the following research question was formulated: are the content and semantics of the instrument for assessing social network support for women with breast cancer undergoing chemotherapy adequate?

## OBJECTIVES

To validate an instrument developed for assessing social network support for women with breast cancer undergoing chemotherapy.

## METHODS

### Ethical aspects

The study was analyzed and approved by the Research Ethics Committee, meeting the requirements of Resolution 466/2012 of the Brazilian National Health Council. All participants (expert judges and women with breast cancer undergoing chemotherapy) were informed about the research objectives, risks, and benefits, and signed an Informed Consent Form (ICF) to participate in the research.

### Study design, period and place

This is a methodological study for the construction and validity of an instrument to assess social network support for women with breast cancer undergoing chemotherapy, which followed these stages: I - Conceptual framework establishment; II - Definition of an instrument’s objectives and the population involved; III - Item and response scale construction; IV - Item selection and organization; V - Instrument structuring; VI - Content validity; and VII - Semantic assessment^(10)^.

The research took place between November 2022 and May 2023. Data collection related to the content validity stage by expert judges, using the nominal group technique (NGT)^(11,12)^, occurred through the use of a virtual environment with the submission of an online form (Google Forms^®^) and via a video call application (Google Meet^®^). Semantic assessment was carried out at the Clinical Oncology Outpatient Clinic of a university hospital located in the city of Recife, Pernambuco, northeastern Brazil.

### Population or sample; inclusion and exclusion criteria

The study population consisted of nurses, who acted as expert judges in analyzing the instrument items and performed its content validity, and women diagnosed with breast cancer undergoing chemotherapy treatment, who participated in the semantic assessment phase of the instrument items.

In the content validity phase, ten expert judges participated, nurses with experience in care, teaching, research and extension activities. Sample size was delimited by the methodological guidelines for the NGT development^(11,12)^, which recommends the participation of between two and ten judges. In the first phase of NGT, all ten judges participated, and in the second phase, two judges. Sampling was carried out using the snowball technique.

The sampling technique involved searching curricula available on the Brazilian National Council for Scientific and Technological Development *Lattes* Platform, using the terms “oncological nursing”, “women’s health nursing”, and “social support”. Upon identifying the judge, the curriculum available on the platform was accessed, and the score of the set of requirements guided by Jasper’s definition of specialists was assessed^(13)^. Achieving at least one point in the requirements triggered contact with judges, via email sent by the platform, inviting them to participate in the survey.

For the semantic assessment phase, women with a biopsy-confirmed diagnosis of breast cancer who had been undergoing chemotherapy treatment at the research site since its inception were included. Women with self-reported cognitive or mental limitations that prevented participation in the research were excluded.

In the semantic assessment stage, the sample consisted of eight women, arranged in two groups: a) four participants with fewer years of education; b) four women with more years of education. Sample size and subdivision of groups followed what is proposed for developing the brainstorming technique, recommended by Pasquali^(10)^, which uses up to eight participants, allocated by stratum from lowest to highest ability. In this study, for this subdivision of participants in this stage, the years of education were chosen to verify their understanding of items in the assessment instrument regarding social network support for women with breast cancer undergoing chemotherapy.

### Study protocol

Data collection followed eight stages in accordance with the framework followed^(10)^. In the first stage, establishing the instrument’s conceptual structure, an integrative literature review study was carried out^(14)^, based on the following research question: what support practices are provided by the social network of women with breast cancer undergoing chemotherapy treatment based on the characteristics of this support? This question was used as a means to gather sources from the Scopus, Web of Science, Medical Literature Analysis and Retrieval System Online, Latin American and Caribbean Literature in Health Sciences, Cumulative Index to Nursing and Allied Health Literature, and Nursing Database databases. The “Social Support”, “Social Networking”, “Drug Therapy”, and “Breast Neoplasms” controlled descriptors were used, as well as the “Chemotherapy” uncontrolled descriptor. The search resulted in 727 publications, which were analyzed according to established eligibility criteria, with nine studies included for synthesis in this review^(14)^.

In addition to gathering information published in databases on the subject, information from books, magazines, printed materials, and grey literature was compiled, allowing for constructing the constitutive and operational dimensions related to the construct and support practices of the social network for women with breast cancer. Lia Sanicola’s Social Network Theory^(5)^ and the methodological guidelines for structuring and validating assessment items provided by Pasquali were used as a theoretical framework^(10)^.

In the second stage, the objective of the instrument for assessing social network support for women with breast cancer undergoing chemotherapy was defined as verifying the support practices offered to women during their treatment experience. It is intended for healthcare professionals as a complementary assessment tool that integrates benefits into care.

In the third stage, item and response scale construction was based on the result of a bibliographic survey, which allowed the identification of dimensions in which support practices are developed (emotional/esteem, companionship, informational, tangible/instrumental, and self-support), as well as the construction of 60 measurement items^(10)^, with responses on a five-point Likert scale (0 - Never; 1 - Almost never; 2 - Sometimes; 3 - Almost always; 4 - Always). In the fourth stage, item selection and organization, the support dimensions offered by the social network were considered, each with its defined purpose, containing aspects related to its function in the practices developed.

In the fifth stage, instrument structuring, the developed items were organized into dimensions in a way that presented the variety of support practices offered by the social network to women with breast cancer during chemotherapy. Items were formulated as statements that women must answer, in order to identify the potential of the support received through the practices developed by members of their social network, considering the behavior, objectivity, simplicity, clarity, precision, relevance, and interpretability criteria^(10)^.

The sixth stage, content validity with expert judges, began after the instrument’s structure was finalized. For content validity, NGT was used, which is a consensus-building method focused on problem exploration and decision-making. It aims to reach a general agreement or convergence of opinion around a specific topic, raising potential solutions or answers to a question, which can then be prioritized or agreed upon. A strength of the consensus method is the balanced participation of group members^(11,12)^.

In this study, NGT was implemented in two phases. Initially, the selected judges received an email invitation to participate in the study. Upon acceptance, an ICF was sent with information related to their participation in the instrument’s content validity process. An online form (Google Forms^®^) containing the instrument items, designed for review and assessment based on the pertinence, clarity, and relevance criteria, was also sent. Space was also included for participants to make suggestions regarding construction of items or their possible exclusion. They were given 15 days to return the completed form.

After the return, a statistical analysis of responses given was carried out, and judges’ suggestions were assessed based on Social Network Theory^(5)^, in order to eliminate or modify the items. Then, in the second phase, a virtual meeting was held, via the Google Meet^®^ platform, with two expert judges^(11,12)^ who had participated in the previous phase, to proceed with the modified instrument content validity, through consensus among these judges, in the final formatting of the instrument. This second phase of NGT was developed in five stages: welcoming and presentation of NGT objectives; silent idea generation; Round Robin; discussion of ideas; and voting for consensus^(11,12)^.

The seventh stage involved content validity completion, which generated the second version of the instrument, and this was submitted to semantic assessment. In this assessment, the women participated in a group brainstorming activity^(10)^, which aimed to present the instrument and request the reproduction of its items through reading and comprehension by participants. Women were divided into two groups: the first group consisted of four participants with fewer years of education; and the second group consisted of four women with more years of education.

In semantic assessment, women, in the defined groups, received the printed instrument to read aloud. For each item read, participants were asked to explain the meaning of the statement expressed in such a way that the other group members agreed or disagreed, generating suggestions for reformulation to better understand the item or its exclusion from the instrument. Participants’ suggestions during the group sessions were recorded and served as a basis for adapting the instrument items when necessary^(10)^.

### Analysis of results and statistics

Judges’ responses were considered, and a database was subsequently created to calculate the Content Validity Coefficient (CVC), which guided the inclusion and exclusion of items from the instrument. A qualitative analysis of suggestions related to item development was also performed, and these were applied as a means of improving the final product. Based on CVC assessment, the Intraclass Correlation Coefficient (ICC) was also calculated to verify the internal consistency of the assessments performed by experts. For CVC and ICC assessment, an index equal to or greater than 80% was established in this study. The items were analyzed individually, and then the instrument as a whole was assessed^(15)^. In semantic assessment, absolute frequencies were considered and Concordance Index (CI) of items was calculated^(15)^.

## RESULTS

The conceptual framework obtained from the literature review aided in formulating the items related to the specificities of social network support practices for women with breast cancer during chemotherapy treatment. The provision of this support was made explicit through actions of an abstract nature (feelings, emotions, and behaviors) and a concrete nature (actions of a practical nature).

This structural format allowed the instrument to be organized into five dimensions, specified by emotional/esteem support, tangible/instrumental support, informational support, companionship support, and self-support. Each dimension included 12 items, which portray the different support practices that can be developed by the members of the social network of a woman with breast cancer during chemotherapy. Therefore, the instrument’s initial version consisted of 60 items.

The content validity assessed by expert judges considered the scores obtained for the items in CVC analysis, representativeness of items in terms of clarity, relevance, and pertinence, as well as their suitability to the reality of caring for women with breast cancer during chemotherapy. The use of NGT for consensus among judges confirmed the findings related to the initial stage of content validity, but did not allow for changes to the instrument’s structure defined in the previous stage.

The content validity phase was conducted by ten judges, all nurses, from all regions of Brazil: Northeast (Pernambuco, Paraíba, Ceará, Bahia, and Alagoas), with five judges, one from each state; Southeast (Rio de Janeiro and São Paulo), with two judges; and North (Amazonas), South (Paraná), and Midwest (Goiás), with one judge each. These participants were between 29 and 43 years old. Four held doctoral degrees, three, master’s degrees, and three, specializations. Their work activities were related to assistance (four), undergraduate teaching (four), healthcare service management (one), and research (one). Judges’ experience in topics related to “women’s health nursing”, “oncology nursing”, and “social support” ranged from five to 18 years.

The mean CVC for each item in relation to clarity of content was 0.944, to pertinence, 0.959, and to theoretical relevance, 0.931. Chart 1 presents the CVC values obtained for each item in the first version of the instrument regarding the clarity, pertinence, and relevance criteria.

**Chart 1:** Content Validity Coefficient of the items in the instrument for assessing social network support (N = 60) for women with breast cancer undergoing chemotherapy, Recife, Pernambuco, Brazil, 2023

Nº	DIMENSION 1 - EMOTIONAL/ESTEEM SUPPORT “Empathy, care, and concern for a person in a stressful situation; positive assessment of a person, expressed through encouragement and agreement with individual ideas and feelings”.	CVC		
		Clarity	Pertinence	Relevance
01	*Sua família se manteve mais próxima de você após o diagnóstico do câncer de mama.*	0.88	0.92	0.94
02	*Seus familiares lhe encorajam/motivam para realização da quimioterapia.*	0.96	1.00	0.98
03	*Sua família se demonstra preocupada com você durante a quimioterapia.*	0.90	1.00	0.96
04	*O relacionamento com o(a) seu(sua) companheiro(a)/esposo(a)/marido/mulher melhorou após o diagnóstico do câncer de mama e início da quimioterapia.*	0.94	0.90	0.88
05	*O relacionamento com os seus filhos melhorou após o diagnóstico do câncer de mama e início da quimioterapia.*	0.88	0.84	0.88
06	*Seus amigos e vizinhos se tornaram mais próximos de você após o diagnóstico do câncer de mama e início da quimioterapia.*	0.90	0.86	0.86
07	*O apoio da sua família ajuda a diminuir o estresse, ansiedade, angústia e medo durante a quimioterapia.*	1.00	0.98	0.98
08	*Você se sente à vontade para expressar seus sentimentos por ser acolhida e ser aconselhada por seus familiares, amigos e vizinhos.*	0.76	0.92	0.92
09	*Após iniciar a quimioterapia, você se sentiu mais valorizada pelas pessoas do seu convívio.*	0.96	0.98	0.92
10	*Seu relacionamento com os colegas de trabalho melhorou após o início da quimioterapia.*	0.98	1.00	0.94
11	*O apoio dado pela sua família lhe faz sentir confiante e confortável para realizar a quimioterapia.*	0.96	0.96	0.94
12	*O apoio que você recebe da sua família, amigos e vizinhos fortalece sua autoestima.*	0.98	0.98	0.90
Nº	**DIMENSION 2 - TANGIBLE/INSTRUMENTAL SUPPORT** “Direct, practical assistance”.	Clarity	Pertinence	Relevance
13	*Você recebe ajuda para se deslocar e realizar as sessões de quimioterapia.*	0.90	0.98	0.92
14	*Sempre vai às sessões de quimioterapia acompanhada de alguém que te ajuda e apoia.*	0.96	0.98	0.94
15	*Recebe ajuda para os seus cuidados quando apresenta reações adversas à quimioterapia.*	0.72	0.98	0.94
16	*Percebe melhora na sua recuperação das reações adversas da quimioterapia quando recebo apoio para se cuidar.*	0.74	0.96	0.90
17	*O apoio que recebe da sua família, amigos, vizinhos e profissionais de saúde melhora a sua qualidade de vida.*	0.98	0.96	0.98
18	*Recebe apoio das pessoas do seu emprego/trabalho para realizar a quimioterapia.*	0.94	0.98	0.94
19	*Recebe apoio dos seus colegas de trabalho durantes os períodos de afastamento do seu emprego.*	0.98	0.96	0.96
20	*Recebe ajuda para realizar os cuidados da sua casa, alimentação, higiene e outras atividades da vida diária durante a quimioterapia.*	0.92	0.96	0.96
21	*Sente que seu estresse e imunidade estão equilibrados quando recebe apoio das pessoas durante a realização da quimioterapia.*	0.78	0.92	0.78
22	*O apoio que recebe das pessoas lhe ajuda a ter tempo para cuidar de você mesma e da sua saúde.*	0.92	0.90	0.92
23	*A ajuda financeira que recebe ajuda na manutenção da qualidade de vida e deixa mais tranquila quanto ao seu sustento e bem-estar.*	0.98	1.00	0.96
24	*O apoio que recebe lhe faz sentir mais protegida/segura durante a quimioterapia.*	0.90	0.96	0.94
Nº	**DIMENSION 3 - INFORMATIONAL SUPPORT** “Offering advice, direction, suggestions, or feedback on how a person is coping with stress”.	Clarity	Pertinence	Relevance
25	*Recebe as informações necessárias e corretas para realizar seus cuidados durante a quimioterapia.*	0.86	0.90	0.94
26	*As informações que recebe sobre seu estado de saúde e tratamento lhe fazem sentir mais confiante e enxergar sua saúde de forma mais positiva.*	0.98	1.00	1.00
27	*Os profissionais que ajudam no seu cuidado estão sempre disponíveis a oferecer informações e tirar suas dúvidas sobre o tratamento.*	0.98	0.98	0.92
Nº	**DIMENSION 3 - INFORMATIONAL SUPPORT** “Offering advice, direction, suggestions, or feedback on how a person is coping with stress”.	Clarity	Pertinence	Relevance
28	*Evita tomar atitudes sobre seu tratamento que não sejam orientadas pelos profissionais de saúde que lhe cuidam durante a quimioterapia.*	0.98	1.00	0.98
29	*Você é incentivada a manter o seguimento das sessões de quimioterapia.*	0.96	1.00	0.98
30	*As informações que recebe sobre o seu tratamento lhe fazem sentir mais confiante durante a quimioterapia.*	1.00	0.96	0.96
31	*Recebe aconselhamento, orientações e informações que usa para seus cuidados no dia a dia.*	0.94	0.96	0.94
32	*Você é incentivada a aplicar as informações que recebe para seu cuidado durante e após a quimioterapia.*	0.94	0.98	0.98
33	*Recebe retorno de como está se saindo nos seus cuidados durante a quimioterapia.*	0.92	0.96	0.92
34	*As informações que recebe lhe ajudam a diminuir o estresse, ansiedade, angústia e o medo durante a quimioterapia.*	0.94	0.96	0.92
35	*As informações que recebe sobre o tratamento ajudam a melhorar o relacionamento com sua família, amigos e vizinhos.*	0.94	0.94	0.86
36	*As informações sobre o seu estado de saúde e seu tratamento são compartilhadas com você de maneira clara e verdadeira.*	0.88	0.88	0.88
Nº	**DIMENSION 4 - COMPANIONSHIP SUPPORT** “Availability to spend time with the person, providing them with feelings of belonging to a group whose members share interests and social activities”.	Clarity	Pertinence	Relevance
37	*Durante as consultas de rotina, exames e sessões de quimioterapia, tem a presença de pessoas que lhe apoiam.*	1.00	0.88	0.86
38	*Se sente encorajada a realizar a quimioterapia quando tem a presença de pessoas que lhe apoiam.*	0.94	0.96	0.94
39	*A presença de pessoas da sua confiança durante a quimioterapia ajuda a diminuir a sensação de náusea, vômito, dores e outros sintomas.*	0.98	0.98	0.96
40	*Sempre tem profissionais disponíveis para lhe ajudar nas dúvidas e necessidades durante a quimioterapia.*	0.96	0.94	0.90
41	*Nos momentos de contato com os profissionais de saúde, sente que tem liberdade e abertura para expressar seus sentimentos e sensações sobre o tratamento e seu estado de saúde.*	0.94	1.00	0.94
42	*Se sente acolhida pelos profissionais quando realiza as sessões de quimioterapia.*	1.00	0.94	0.94
43	*Durante as sessões de quimioterapia, fez novos amigos que lhe apoiam durante o tratamento.*	0.98	1.00	0.96
44	*É ouvida quando tem dúvidas, angústias e preocupações sobre o seu estado de saúde e tratamento.*	0.94	0.96	0.94
45	*Recebe cuidados de pessoas próximas quando não consegue se cuidar sozinha.*	0.98	0.98	0.96
46	*Participa de atividades em grupo que lhe ajudam a se sentir mais confiante durante a quimioterapia.*	0.98	0.96	0.94
47	*Se sente segura por ter a presença de pessoas que cuidam de você durante as sessões de quimioterapia.*	0.96	0.96	0.94
48	*Se sente motivada a participar de atividades sociais com pessoas próximas a você.*	0.98	0.98	0.92
Nº	**DIMENSION 5 - SELF-SUPPORT** “Personal motivation for decision-making, intimate and personal intentions related to the situations experienced”.	Clarity	Pertinence	Relevance
49	*Se sente motivada a realizar as sessões de quimioterapia.*	1.00	0.96	0.96
50	*Se sente segura para tomar as decisões sobre seu tratamento.*	1.00	0.98	0.96
51	*Sente que seu humor e motivação para as atividades de lazer e convívio social se mantêm equilibrados durante a quimioterapia.*	0.98	0.94	0.94
52	*Busca informações sobre a quimioterapia como forma de entender melhor como esta acontece e se sentir mais encorajada.*	0.98	0.98	0.96
53	*Tem pensamentos, atitudes e expectativas positivas sobre realizar o tratamento com quimioterapia.*	0.98	0.98	0.96
54	*Busca motivação na sua família, amigos e religião para superar os sentimentos de tristeza, decepção e dor.*	1.00	0.98	0.96
55	*Os efeitos adversos da quimioterapia são passageiros, e pensar sobre isso lhe faz sentir mais animada para finalizar o tratamento.*	0.94	0.94	0.84
56	*Compreende que as mudanças que acontecem no seu corpo durante a quimioterapia não são permanentes e não afetam sua autoestima.*	0.96	0.98	0.88
57	*Melhorou seu entendimento sobre a quimioterapia após passar pelo tratamento.*	0.98	0.98	0.90
Nº	**DIMENSION 5 - SELF-SUPPORT** “Personal motivation for decision-making, intimate and personal intentions related to the situations experienced”.	Clarity	Pertinence	Relevance
58	*Se sente motivada a cuidar do meu corpo, aparência física e autoestima.*	1.00	0.96	0.94
59	*Busca na sua prática religiosa/espiritual motivação para enfrentar as sessões de quimioterapia.*	1.00	0.98	0.96
60	*Procura manter contato com amigos, vizinhos e família para apoio no enfrentamento da quimioterapia.*	1.00	1.00	0.98

*CVC - Content Validity Coefficient.*

The results of ICC were similar to those estimated by the total CVC, with agreement of 0.918 for the instrument’s content clarity criterion, 0.957 for the practical relevance criterion, and 0.967 for the theoretical relevance criterion, where all coefficients were statistically significant. The interpretation of ICC is related to the agreement of judges’ scores, with ICC equal to zero when there is no agreement in the scores among evaluators, and equal to one when there is total agreement in the scores among evaluators. It was observed that, in all items, there was almost perfect agreement (between 0.8 and 1.00) in all domains assessed in the instrument, as shown in Table 1.

**Table 1:** Total Content Validity Coefficient and Intraclass Correlation Coefficient according to domains in the language clarity, practical pertinence, and theoretical relevance dimensions, Recife, Pernambuco, Brazil, 2023

Domains	Mean (95% CI)^*^	*p* value
**Total Content Validity Coefficient**		
Content clarity	0.944 (0.928 - 0.959)^a^	-
Practical pertinence	0.959 (0.949 - 0.969)^a^	-
Theoretical relevance	0.931 (0.920 -0.941)^b^	-
**Intraclass Correlation Coefficient**		
Content clarity	0.918 (0.825 - 0.975)^a^	<0.001
Practical pertinence	0.957 (0.909 - 0.987)^a^	<0.001
Theoretical relevance	0.967 (0.930 - 0.990)^a^	<0.001

* *For the difference between domains, for identical letters, there was no significant difference (p>0.05); 95% CI - 95% Confidence Interval.*

Based on statistical analysis and judges’ considerations regarding the writing and elaboration of the instrument items, the following were excluded: items 05 and 21 (CVC <0.80 in at least one of the analyzed criteria); items 06, 09, 14, 28, 29, and 40 (the practice of support was already contemplated in another item of the instrument); item 17 (did not express concrete help consistent with the tangible/instrumental support dimension); and items 10, 18, 19, and 46 (support practice linked to a specific life condition that women undergoing chemotherapy treatment might not experience). Items 8, 15, and 16 presented a CVC <0.80 only in the clarity criterion. In the second phase of NGT, because they were pertinent and relevant items (CVC>80) for the instrument, judges retained them with improvements in their writing (Chart 1). After eliminations, the second version of the instrument had 47 items, which were submitted to semantic assessment with the target audience.

The eight women participating in semantic assessment were between 37 and 61 years old, with a mean age of 48, and all stated they were married. Regarding their years of education, it was observed that the educational background of these women ranged from incomplete primary education to completed higher education.

In this assessment, the mean CI was 0.99 points. Some participants had difficulty understanding items 16, 19, 35, and 46. However, these items were not excluded, as the authors chose to retain them due to their suitability in assessing the support offered by the social network of women with breast cancer and the satisfactory CI values obtained. It should be noted, however, that the suggestions were accepted, and the wording of these items was adjusted for clarity.

Upon completion of these stages, the third version of the instrument for assessing social support for women with breast cancer undergoing chemotherapy was finalized. This final version (Appendix) consists of 47 items, distributed among the following support dimensions: emotional/esteem (eight items); tangible/instrumental (seven); informational (10); emotional/esteem (10); and self-support (12 items).

## DISCUSSION

The items in the instrument were developed using a theoretical construct related to social network support practices for women with breast cancer undergoing chemotherapy treatment, reflecting the characteristics of support found in the literature, especially in publications from Asia (Thailand, South Korea, and Japan), Europe (Sweden), and the Americas (Brazil and the United States)^(16-24)^. In their records, the authors highlighted social network practices that can improve women’s well-being and quality of life throughout their treatment^(14,16-24)^.

The review phase, which encompassed studies from various continents, made it possible to establish the concept of the study’s object (social network support practices for women with breast cancer undergoing chemotherapy), its constitutive and operational attributes, which guided the development of the instrument items in order to operationalize the construct into items upon completion^(25)^.

The development and validity of an instrument aimed at assessing social network support represents a significant advancement in healthcare, especially in oncology. In the context of assisting women with breast cancer undergoing chemotherapy, enhancing professional practice with non-procedural actions allows for comprehensive care that is tailored to the individual needs of each person^(26)^.

The validity stages, based on recommendations from judges and the target audience, produced a suitable and reliable instrument for use by healthcare professionals and the scientific community to improve clinical practice and investigate the social support offered to women with breast cancer undergoing chemotherapy. Despite its importance, obstacles were encountered in ensuring the effective participation of judges. This difficulty, stemming from the loss of judges, is a concern in validity studies, which are methodical and require significant time contributions^(10)^.

In the content validity phase, it was observed that judges’ academic qualifications were more heavily emphasized, with doctoral degrees, academic and clinical experience, which conferred greater reliability at this stage^(11,12)^. The use of NGT favored the instrument’s suitability by generating tangible results within a relatively short timeframe. However, it was observed that, even though it is viable for use in the content validity process, it requires planning and execution within a short timeframe so that the instrument’s particularities are not diluted and, consequently, this does not result in losses in consensus among judges^(11,12)^.

Judges’ contributions to the item design were relevant for organizing the statements in the support dimensions structured from the construct, especially in the reformulation of items that presented a CVC related to clarity lower than the cut-off point established in the method (items 8, 15, and 16). The suggestion to eliminate items 5, 6, 9, 10, 14, 28, 29, and 40 was accepted because the essence of the content of these items had already been addressed in other statements. Item 21 was eliminated because the CVC result was unsatisfactory. Item eliminations also occurred in other studies^(25,27)^, since there was similarity in the elaboration of items in different dimensions, due to the theoretical deepening of the construct and the analyzed coefficients not being significant.

Support from colleagues is important in the process of coping with changes resulting from treatment, strengthening the sense of belonging to a group, self-esteem, and providing acceptance^(5)^. Despite this, items 10, 18, and 19, which dealt with primary care network support related to the development of work activities during chemotherapy, were excluded because, during the questioning, the female study participant might not have been able to answer due to not working or being on sick leave.

Evidence shows that absenteeism from work among women with breast cancer is increasing, whether due to the Social Security Professional Rehabilitation Program, job abandonment due to worsening health conditions, or experiencing prejudice and other negative experiences^(28)^.

Item 46, concerning group activities for women undergoing chemotherapy, was suggested for elimination by judges, as they may not be suitable candidates for such activities. Chemotherapy can cause hematological toxicity, leading to negative impacts on a woman’s life, potentially resulting in reduced immunity due to decreased blood cell counts, and potentially leading to leukopenia, thrombocytopenia, febrile neutropenia, and anemia^(29)^. Therefore, social activities in groups and communities should be restricted when these conditions occur, as they present a risk of comorbidities during treatment^(30)^.

Semantic assessment allowed for modifications related to the wording of some words/terms in items 16, 19, 35, and 46, aiming for a better understanding of the questions posed by the target audience for whom the instrument is intended. The suggestions were accepted and incorporated into the final version. This stage can be understood as an important advance for translation of nursing knowledge, especially in studies that develop technologies for care. The literature highlights a growing concern with conducting research with patients in real-world contexts, with the goal of increasing standardized language systems and promoting their translation into practice^(31)^.

The criteria adopted for content validity and semantic assessment in this study were based on references that allowed a thorough assessment of item reliability and production of a reliable and appropriate instrument for use in the care of women with breast cancer. The process followed for the validity of an instrument is crucial in the application of the measure for which it is intended^(10)^. It is fundamental to carry out validity stages to determine a study results’ legitimacy and credibility and the recognition of the quality of an instrument’s items^(32)^.

### Study limitations

Although meeting the sample size recommended by the literature^(10)^, the study was considered limited by the number of judges who collaborated in the second phase of NGT, since a larger number of judges would allow for a more thorough and exhaustive discussion of the instrument, in order to further improve the product constructed in this study.

### Contributions to health, nursing, or public policy

The use of this instrument in the clinical practice of nurses focused on the care of women undergoing chemotherapy provides parameters for assessing the support provided by the woman’s social network. This assessment can support actions to mobilize the primary and secondary social network, in order to implement necessary interventions so that the support offered by the social network of women with breast cancer translates into real benefits in coping with this neoplasm. Furthermore, this validated instrument can be applied in teaching strategies in undergraduate nursing courses, as well as in scientific research on women with breast cancer undergoing chemotherapy.

## CONCLUSIONS

The instrument made available in this study to oncology professionals is capable of assessing social network support for women with breast cancer during chemotherapy follow-up. Its construction and validity process, based on theoretical and methodological frameworks established in the literature, demonstrates that this tool has been validated, is safe, and is suitable for its intended purpose.

The conceptual framework definition allowed for the identification of social network support practices, aligning them with the specific experiences of women with breast cancer during chemotherapy in the instrument item construction. The identification of content validity evidence ensured item adequacy in terms of clarity, pertinence, and relevance, making the instrument reliable for professionals to safely investigate the phenomenon of social network support in their daily care. Semantic assessment, in turn, provided clarity of wording for the target audience to understand the instrument items. However, a clinical validity study of the instrument is recommended.

## Data Availability

The research data are available within the article.
