# Supplementary material for: Instrument for assessing social network support for women with breast cancer
Source: Rev Bras Enferm. 2026 Jul 27;79:e20250148. doi: 10.1590/0034-7167-2025-0148 (PMC13411315; doi:10.1590/0034-7167-2025-0148)
Supplement: Translation

# Translation: Instrumento de avaliação do apoio da rede social à mulher com câncer de mama

**DOI:** 10.1590/0034-7167-2025-0148pt

**Authors:** Diego Augusto Lopes Oliveira, Vânia Pinheiro Ramos, Luciana Pedrosa Leal, Gabriela Cunha Schechtman Sette, Wilson Jorge Correia Pinto de Abreu, Cleide Maria Pontes

**Affiliations:** I Universidade de Pernambuco. Recife, Pernambuco, Brasil; II Universidade Federal de Pernambuco. Recife, Pernambuco, Brasil; III Escola Superior de enfermagem do Porto. Porto, Portugal

**Keywords:** Neoplasias da Mama, Estudo de Validação, Tratamento Farmacológico, Rede Social, Apoio Social.

## Abstract

**Objetivos:**

validar instrumento para avaliação o apoio da rede social às mulheres com câncer de mama em quimioterapia.

**Métodos:**

estudo metodológico com etapas de construção baseadas na Teoria das Redes Sociais. Realizaram-se validade de conteúdo, por dez juízes (grupo nominal), e avaliação semântica, por oito mulheres (*brainstorming*). Os dados foram analisados usando frequências relativas, Coeficiente de Validade de Conteúdo, Coeficiente de Correlação Intraclasse e Índice de Confiança.

**Resultados:**

o instrumento com cinco dimensões de apoio (emocional, informativo, instrumental, presencial e autoapoio) possuía 60 itens. A maioria tinha coeficientes ≥0,80 na avaliação dos juízes. Após eliminar itens e realizar ajustes, o instrumento ficou com 47 itens, com avaliação semântica que alcançou Índice de Confiança de 0,99. Realizaram-se ajustes para maior clareza, mantendo-se os 47 itens nas cinco dimensões do apoio.

**Conclusões:**

validou-se o instrumento como ferramenta para avaliar o apoio da rede social às mulheres com câncer de mama em quimioterapia.

## INTRODUÇÃO

O câncer de mama é a neoplasia que mais leva mulheres a óbito no mundo, com notificação de cerca de 2,3 milhões de mortes no ano de 2020^(1)^. O aumento no número de casos está atrelado ao envelhecimento populacional, alterações nos padrões de consumo, estilo de vida e políticas públicas frágeis relacionadas ao diagnóstico precoce e rastreio da doença^(2)^. Tal panorama impede o desenrolar de uma linha de cuidado que permita melhores condições para tratamento e prognóstico das mulheres acometidas^(1,2)^.

As estratégias estabelecidas para abordagem e intervenção no manejo do câncer mamário destacam a quimioterapia como tratamento que otimiza o tempo e retarda o avanço tumoral. Sua utilização implica a administração de fármacos que produzem efeitos tóxicos ao DNA celular. Também o seu uso traz consequências à mulher na vivência de reações adversas que implicam mudanças no seu contexto físico e mental, na qualidade de vida e interação social, especialmente na manutenção dos relacionamentos e no apoio recebido pela rede social^(3,4)^.

A rede social de uma pessoa se constitui a partir de vínculos estabelecidos no seu cotidiano, configurando-se pelos laços de relacionamento que promovem parceria, troca e intercâmbio de apoio social. A oferta de apoio da rede social nas dimensões emocional (comportamentos de empatia, carinho e preocupação com a mulher), presencial (disponibilidade de tempo para acompanhar a mulher, ser amparo e proporcionar sentimento de pertencimento a um grupo social), informativa (conselhos, orientações, sugestões e retorno do comportamento da mulher diante do enfrentamento das adversidades impostas pelo adoecimento), instrumental (ajuda prática em substituição a atividades da vida diária que não podem ser desenvolvidas em virtude do adoecimento) e de autoapoio (decisões individuais da mulher e motivação para enfrentamento dos tratamentos, adversidades da doença e adesão às propostas de melhoria da sua saúde) permite fortalecimento do enfrentamento do câncer e da quimioterapia pela mulher^(5-7)^.

Os relacionamentos sociais da mulher diagnosticada com câncer de mama sofrem transformações significativas ao longo da quimioterapia, ora tornando seus laços sociais fragilizados, ora fortalecendo-os. Esse movimento está relacionado diretamente ao apoio fornecido pela rede social, de modo que sua avaliação se faz necessária para que medidas que fortaleçam as práticas de apoio seja demandadas e culminem em benefícios no processo de cuidado ao longo do uso de antineoplásicos^(8)^.

A avaliação do apoio da rede social à mulher com câncer de mama durante a quimioterapia é uma estratégia que colabora com o cuidado integral, através de ações não procedimentais que envolvem família, amigos, vizinhos e profissionais de saúde na promoção do bem-estar, qualidade de vida e superação de situações difíceis vividas durante a jornada de enfrentamento de uma neoplasia^(5)^.

A construção e validade de tecnologias de cuidado, especialmente de instrumentos de avaliação, têm sido cada vez mais utilizadas como forma de propor a apresentação de resultados cientificamente robustos acerca das ações de cuidado de pessoas, famílias e comunidades. Ressalta-se a importância de cumprimento do rigor teórico e metodológico para alcance de um instrumento apropriado que se propõe a mensurar/avaliar e que possua critérios na construção dos itens de avaliação^(9)^. Diante desse contexto, formulou-se a seguinte pergunta de pesquisa: o conteúdo e a semântica do instrumento de avaliação do apoio da rede social às mulheres com câncer de mama em quimioterapia são adequados?

## OBJETIVOS

Validar instrumento construído para avaliação do apoio da rede social às mulheres com câncer de mama em quimioterapia.

## MÉTODOS

### Aspectos éticos

O estudo foi analisado e aprovado pelo Comitê de Ética em Pesquisa, atendendo às prerrogativas da Resolução nº 466/2012 do Conselho Nacional de Saúde. Todos os participantes (juízes especialistas e mulheres com câncer de mama em tratamento quimioterápico) foram instruídos quanto aos objetivos, riscos e benefícios da pesquisa, e assinaram Termo de Consentimento Livre e Esclarecido (TCLE) para contribuição na pesquisa.

### Desenho, período e local do estudo

Estudo metodológico para construção e validade de instrumento para avaliação do apoio da rede social às mulheres com câncer de mama em quimioterapia, no qual se percorreram as seguintes etapas: I - Estabelecimento da estrutura conceitual; II - Definição dos objetivos do instrumento e da população envolvida; III - Construção dos itens e das escalas de resposta; IV - Seleção e organização dos itens; V - Estruturação do instrumento; VI - Validade de conteúdo; e VII - Avaliação semântica^(10)^.

A pesquisa ocorreu no período de novembro de 2022 a maio de 2023. A coleta dos dados relacionada à etapa de validade de conteúdo pelos juízes especialistas, por meio da técnica de grupo nominal (TGN)^(11,12)^, ocorreu através do uso de ambiente virtual com envio de formulário *online* (*Google Forms^®^*) e por aplicativo de videochamadas (*Google Meet^®^*). A avaliação semântica foi realizada no Ambulatório de Oncologia Clínica de hospital universitário localizado na cidade de Recife, Pernambuco, nordeste do Brasil.

### População ou amostra; critérios de inclusão e exclusão

A população do estudo foi composta por enfermeiros, que atuaram como juízes especialistas na análise dos itens do instrumento e realizaram sua validação de conteúdo, e por mulheres diagnosticadas com câncer de mama em realização do tratamento quimioterápico, que participaram da etapa de avaliação semântica dos itens do instrumento.

Na etapa de validade de conteúdo, participaram dez juízes especialistas, enfermeiros com experiência em atividades assistenciais, de ensino, de pesquisa e de extensão. O tamanho amostral foi delimitado pela orientação metodológica para o desenvolvimento da TGN^(11,12)^, que preconiza a participação entre dois e dez juízes. Na primeira etapa da TGN, participaram os dez juízes, e na segunda etapa, dois juízes. A amostragem ocorreu pela técnica de *snowball*.

A técnica de amostragem ocorreu a partir da busca dos currículos disponibilizados na Plataforma *Lattes* do Conselho Nacional de Desenvolvimento Científico e Tecnológico, por meio do uso dos termos “enfermagem oncológica”, “enfermagem em saúde da mulher” e “apoio social”. Na identificação do juiz, foi acessado o currículo disponível na plataforma, sendo avaliada a pontuação do conjunto de requisitos orientados pela definição de especialistas recomendada por Jasper^(13)^. O alcance de pelo menos um ponto nos requisitos orientou o contato com o juiz, através de e-mail enviado pela plataforma, convidando-o a participar da pesquisa.

Para etapa de avaliação semântica, foram incluídas mulheres com diagnóstico de câncer de mama confirmado por biópsia e que estivessem em seguimento do tratamento com quimioterapia no local da pesquisa desde o seu início. Foram excluídas mulheres que apresentassem limitações cognitivas ou mentais, autorreferidas, que fossem impeditivas da participação na pesquisa.

Na etapa de avaliação semântica, a amostra foi composta por oito mulheres, dispostas em dois grupos: a) quatro participantes com menor número de anos de estudo; b) quatro mulheres com maior número de anos de estudo. O quantitativo amostral e a subdivisão de grupos definidos seguiram o que é proposto para o desenvolvimento da técnica de *brainstorming*, recomendada por Pasquali^(10)^, que utiliza até oito participantes, alocados por extrato de mais baixa ao de maior habilidade. Neste estudo, para esta subdivisão das participantes dessa etapa, optaram-se pelos anos de estudo para verificar a compreensão dos itens do instrumento de avaliação do apoio da rede social às mulheres com câncer de mama em tratamento quimioterápico.

### Protocolo do estudo

A coleta de dados percorreu as oito etapas em consonância com o referencial seguido^(10)^. Na primeira etapa, estabelecimento da estrutura conceitual do instrumento, realizou-se estudo de revisão integrativa da literatura^(14)^, alicerçado na seguinte pergunta de pesquisa: quais as práticas de apoio fornecidas pela rede social de mulheres com câncer de mama em tratamento quimioterápico alicerçadas nas características desse apoio? Esta questão foi utilizada como meio para levantamento de fontes nas bases de dados Scopus, *Web of Science, Medical Literature Analysis and Retrieval System Online*, Literatura Latino-Americana e do Caribe em Ciências da Saúde, *Cumulative Index to Nursing and Allied Health Literature* e Banco de Dados em Enfermagem. Utilizaram-se os descritores controlados, *“Social Support”, “Social Networking”, “Drug Therapy”, “Breast Neoplasms”*, e o não controlado *“Chemoterapy”*. A pesquisa resultou em 727 publicações, que foram analisadas conforme os critérios de elegibilidade estabelecidos, sendo inclusos nove estudos para síntese da revisão^(14)^.

Além do levantamento das informações publicadas nas bases de dados acerca da temática, foram elencadas informações de livros, revistas, materiais impressos e da literatura cinzenta, que permitiram a construção das dimensões constitutivas e operacionais relativas ao construto e práticas de apoio da rede social às mulheres com câncer de mama. Utilizaram-se como referencial teórico a Teoria das Redes Sociais de Lia Sanicola^(5)^ e as orientações metodológicas para estruturação e validade de itens de avaliação orientados por Pasquali^(10)^.

Na segunda etapa, o objetivo do instrumento de avaliação do apoio da rede social às mulheres com câncer de mama em quimioterapia foi definido para verificar as práticas de apoio ofertadas à mulher durante a vivência do tratamento. Destina-se a profissionais de saúde como ferramenta de avaliação complementar à assistência que integra benefícios ao cuidado.

Na terceira etapa, e construção dos itens e da escala de resposta se baseou no resultado do levantamento bibliográfico, que permitiu a identificação das dimensões nos quais são desenvolvidas as práticas de apoio (apoio emocional, apoio presencial, apoio informativo, apoio instrumental e autoapoio), bem como a construção de 60 itens de aferição^(10)^, com respostas em escala Likert de cinco pontos (0 - Nunca; 1 - Quase nunca; 2 - Às vezes; 3 - Quase sempre; 4 - Sempre). Na quarta etapa, seleção e organização dos itens, consideraram-se as dimensões de oferta do apoio pela rede social, cada uma com sua finalidade definida, contendo os aspectos relacionados à sua função nas práticas desenvolvidas.

Na quinta etapa, estruturação do instrumento, os itens elaborados foram organizados nas dimensões de forma que se apresentasse a variedade das práticas de apoio da rede social às mulheres com câncer de mama durante a quimioterapia. Os itens foram formulados por meio de afirmações, que devem ser respondidas pela mulher, de maneira a identificar o potencial do apoio recebido através da prática desenvolvida pelos membros de sua rede social, considerando os critérios comportamentais, de objetividade, de simplicidade, de clareza, de precisão, de relevância e de interpretabilidade^(10)^.

A sexta etapa, validade de conteúdo com os juízes especialistas, foi iniciada após a finalização da estrutura do instrumento. Para validade de conteúdo, utilizou-se a TGN, que consiste em um método de consenso com foco na exploração de problemas e tomada de decisão. Visa alcançar um acordo geral ou convergência de opinião em torno de um tópico específico, levantando soluções ou respostas potenciais para uma questão, que pode então ser priorizada ou acordada. Um ponto forte do método de consenso é a participação equilibrada dos membros do grupo^(11,12)^.

Neste estudo, a TGN foi operacionalizada em dois momentos. Inicialmente, os juízes selecionados receberam via e-mail o convite para participação do estudo. Após o aceite, foi enviado TCLE com as informações relacionadas à sua participação no processo de validade de conteúdo do instrumento. Também foi enviado formulário *online* (*Google Forms^®^*), contendo os itens do instrumento, elaborado para apreciação e avaliação mediante os critérios de pertinência, clareza e relevância. Foi incluído, ainda, espaço para que os participantes realizassem suas sugestões na construção dos itens ou na sua possível exclusão. Foram dados 15 dias para devolução do formulário preenchido.

Após a devolução, foi feita análise estatística das respostas dadas, e as sugestões dos juízes foram avaliadas com base na Teoria das Redes Sociais^(5)^, no sentido de eliminar ou modificar os itens. Em seguida, no segundo momento, foi realizado encontro virtual, através da plataforma *Google Meet^®^*, com dois juízes especialistas^(11,12)^ participantes do momento anterior, para proceder à validade de conteúdo do instrumento modificado, por meio de consenso entre esses juízes, na formatação final do instrumento. Este segundo momento da TGN foi desenvolvido em cinco fases: acolhimento e apresentação dos objetivos da TGN; geração silenciosa de ideias; *Round Robin;* discussão das ideias; e votação para consenso^(11,12)^.

A sétima etapa ocorreu com a conclusão da validade de conteúdo, que gerou a segunda versão do instrumento, e esta foi submetida à avaliação semântica. Nessa avaliação, as mulheres participaram de atividade de grupo do tipo *brainstorming* ^(10)^, que tinha como objetivo apresentar o instrumento e solicitar a reprodução dos seus itens mediante a leitura e compreensão das participantes. As mulheres foram dispostas em dois grupos: o primeiro possui quatro participantes com menor número de anos de estudo; e o segundo possui quatro mulheres com maior número de anos de estudo.

Na avaliação semântica, as mulheres, nos grupos definidos, receberam o instrumento impresso para leitura em voz alta. A cada item lido, as participantes foram solicitadas a explicitar o significado da afirmação expressa de forma que os demais membros do grupo concordassem ou discordassem a ponto de gerar sugestões de reformulação para melhor compreensão do item ou sua exclusão do instrumento. As sugestões das participantes durante o grupo foram registradas e serviram de base para adequação dos itens do instrumento, quando necessário^(10)^.

### Análise dos resultados e estatística

As respostas dos juízes foram consideradas, sendo posteriormente elaborado banco de dados para cálculo do Coeficiente de Validade de Conteúdo (CVC), que norteou a inclusão e exclusão dos itens do instrumento. Foi realizada, ainda, análise qualitativa das sugestões relacionadas à elaboração dos itens, sendo essas aplicadas como meio de melhoramento do produto final. Mediante a avaliação do CVC, foi realizado, ainda, o cálculo do Coeficiente de Correlação Intraclasse (CCI) para verificação da consistência interna das avaliações realizadas pelos especialistas. Para avaliação do CVC e do CCI, foi estabelecido, neste estudo, um índice igual ou superior a 80%. Os itens foram analisados de forma individual, e depois ocorreu avaliação do instrumento como um todo^(18)^. Na avaliação semântica, foram consideradas as frequências relativas e calculado o Índice de Concordância (IC) dos itens^(15)^.

## RESULTADOS

A estrutura conceitual obtida, a partir do levantamento da literatura, auxiliou na formulação dos itens atrelados às particularidades das práticas de apoio da rede social às mulheres com câncer de mama durante o tratamento quimioterápico. A oferta desse apoio foi explicitada a partir de ações de característica abstrata (sentimentos, emoções e comportamentos) e concreta (ações de natureza prática).

Essa formatação estrutural possibilitou a organização do instrumento em cinco dimensões, especificadas pelo apoio emocional, apoio instrumental, apoio informativo, apoio presencial e autoapoio. Cada dimensão contemplou 12 itens, que retratam as diferentes práticas de apoio que podem ser desenvolvidas pelos componentes da rede social da mulher com câncer de mama durante a quimioterapia. Portanto, a versão inicial do instrumento foi composta por 60 itens.

A validade de conteúdo pelos juízes especialistas levou em consideração os índices obtidos pelos itens na análise do CVC, a representatividade dos itens no que tange à clareza, relevância e pertinência, bem como a adequação dos mesmos à realidade do cuidar da mulher com câncer de mama durante a quimioterapia. O uso da TGN para consenso dos juízes confirmou os achados relacionados à etapa inicial da validade de conteúdo, porém não oportunizou mudanças na estrutura do instrumento definida na etapa anterior.

A etapa de validade de conteúdo foi realizada por dez juízes, todos enfermeiros, de todas as regiões do Brasil: Nordeste (Pernambuco, Paraíba, Ceará, Bahia e Alagoas), com cinco juízes, sendo um de cada estado; Sudeste (Rio de Janeiro e São Paulo), com dois juízes; e Norte (Amazonas), Sul (Paraná) e Centro-Oeste (Goiás), com um juiz cada. Esses participantes estavam na faixa etária entre 29 e 43 anos. Quatro possuíam doutorado, três, mestrado, e três, especialização. As atividades laborais desenvolvidas por eles eram relacionadas à assistência (quatro), ensino de graduação (quatro), gestão de serviços de saúde (um) e pesquisa (um). A experiência dos juízes nas temáticas relacionadas à “enfermagem em saúde da mulher”, “enfermagem oncológica” e “apoio social” variou entre cinco e 18 anos de atuação.

A média dos CVCs de cada item em relação à clareza de conteúdo foi de 0,944, à pertinência, de 0,959, e à relevância teórica, de 0,931. Os valores de CVCs obtidos em cada item da primeira versão do instrumento quanto aos critérios de clareza, pertinência e relevância estão apresentados no Quadro 1.

**Quadro 1:** Coeficiente de Validade de Conteúdo dos itens do instrumento de avaliação do apoio da rede social (N = 60) às mulheres com câncer de mama em tratamento quimioterápico, Recife, Pernambuco, Brasil, 2023

Nº	DIMENSÃO 1 - APOIO EMOCIONAL *(Emotional/Esteem Support)* “Empatia, carinho e preocupação em relação à pessoa em situação de estresse; valorização positiva da pessoa, que se expressa no encorajamento e concordância com ideias e sentimentos individuais”.	CVC		
		Clareza	Pertinência	Relevância
01	Sua família se manteve mais próxima de você após o diagnóstico do câncer de mama.	0,88	0,92	0,94
02	Seus familiares lhe encorajam/motivam para realização da quimioterapia.	0,96	1,00	0,98
03	Sua família se demonstra preocupada com você durante a quimioterapia.	0,90	1,00	0,96
04	O relacionamento com o(a) seu(sua) companheiro(a)/esposo(a)/marido/mulher melhorou após o diagnóstico do câncer de mama e início da quimioterapia.	0,94	0,90	0,88
05	O relacionamento com os seus filhos melhorou após o diagnóstico do câncer de mama e início da quimioterapia.	0,88	0,84	0,88
06	Seus amigos e vizinhos se tornaram mais próximos de você após o diagnóstico do câncer de mama e início da quimioterapia.	0,90	0,86	0,86
07	O apoio da sua família ajuda a diminuir o estresse, ansiedade, angústia e medo durante a quimioterapia.	1,00	0,98	0,98
08	Você se sente à vontade para expressar seus sentimentos por ser acolhida e ser aconselhada por seus familiares, amigos e vizinhos.	0,76	0,92	0,92
09	Após iniciar a quimioterapia, você se sentiu mais valorizada pelas pessoas do seu convívio.	0,96	0,98	0,92
10	Seu relacionamento com os colegas de trabalho melhorou após o início da quimioterapia.	0,98	1,00	0,94
11	O apoio dado pela sua família lhe faz sentir confiante e confortável para realizar a quimioterapia.	0,96	0,96	0,94
12	O apoio que você recebe da sua família, amigos e vizinhos fortalece sua autoestima.	0,98	0,98	0,90
Nº	**DIMENSÃO 2 - APOIO INSTRUMENTAL** ***(Tangible/Instrumental support*)** “Ajuda direta, de natureza prática”.	Clareza	Pertinência	Relevância
13	Você recebe ajuda para se deslocar e realizar as sessões de quimioterapia.	0,90	0,98	0,92
14	Sempre vai às sessões de quimioterapia acompanhada de alguém que te ajuda e apoia.	0,96	0,98	0,94
15	Recebe ajuda para os seus cuidados quando apresenta reações adversas à quimioterapia.	0,72	0,98	0,94
16	Percebe melhora na sua recuperação das reações adversas da quimioterapia quando recebo apoio para se cuidar.	0,74	0,96	0,90
17	O apoio que recebe da sua família, amigos, vizinhos e profissionais de saúde melhora a sua qualidade de vida.	0,98	0,96	0,98
18	Recebe apoio das pessoas do seu emprego/trabalho para realizar a quimioterapia.	0,94	0,98	0,94
19	Recebe apoio dos seus colegas de trabalho durantes os períodos de afastamento do seu emprego.	0,98	0,96	0,96
20	Recebe ajuda para realizar os cuidados da sua casa, alimentação, higiene e outras atividades da vida diária durante a quimioterapia.	0,92	0,96	0,96
21	Sente que seu estresse e imunidade estão equilibrados quando recebe apoio das pessoas durante a realização da quimioterapia.	0,78	0,92	0,78
22	O apoio que recebe das pessoas lhe ajuda a ter tempo para cuidar de você mesma e da sua saúde.	0,92	0,90	0,92
23	A ajuda financeira que recebe ajuda na manutenção da qualidade de vida e deixa mais tranquila quanto ao seu sustento e bem-estar.	0,98	1,00	0,96
24	O apoio que recebe lhe faz sentir mais protegida/segura durante a quimioterapia.	0,90	0,96	0,94
Nº	**DIMENSÃO 3 - APOIO INFORMATIVO** ***(Informational support)*** “Oferta de conselhos, direções, sugestões ou retorno de como a pessoa está se saindo no enfrentamento do estresse”.	Clareza	Pertinência	Relevância
25	Recebe as informações necessárias e corretas para realizar seus cuidados durante a quimioterapia.	0,86	0,90	0,94
26	As informações que recebe sobre seu estado de saúde e tratamento lhe fazem sentir mais confiante e enxergar sua saúde de forma mais positiva.	0,98	1,00	1,00
27	Os profissionais que ajudam no seu cuidado estão sempre disponíveis a oferecer informações e tirar suas dúvidas sobre o tratamento.	0,98	0,98	0,92
28	Evita tomar atitudes sobre seu tratamento que não sejam orientadas pelos profissionais de saúde que lhe cuidam durante a quimioterapia.	0,98	1,00	0,98
29	Você é incentivada a manter o seguimento das sessões de quimioterapia.	0,96	1,00	0,98
30	As informações que recebe sobre o seu tratamento lhe fazem sentir mais confiante durante a quimioterapia.	1,00	0,96	0,96
31	Recebe aconselhamento, orientações e informações que usa para seus cuidados no dia a dia.	0,94	0,96	0,94
32	Você é incentivada a aplicar as informações que recebe para seu cuidado durante e após a quimioterapia.	0,94	0,98	0,98
33	Recebe retorno de como está se saindo nos seus cuidados durante a quimioterapia.	0,92	0,96	0,92
34	As informações que recebe lhe ajudam a diminuir o estresse, ansiedade, angústia e o medo durante a quimioterapia.	0,94	0,96	0,92
35	As informações que recebe sobre o tratamento ajudam a melhorar o relacionamento com sua família, amigos e vizinhos.	0,94	0,94	0,86
36	As informações sobre o seu estado de saúde e seu tratamento são compartilhadas com você de maneira clara e verdadeira.	0,88	0,88	0,88
Nº	**DIMENSÃO 4 - APOIO PRESENCIAL** **(*Companionship support*)** “Disponibilidade para passar tempo com a pessoa, proporcionando-lhe sentimentos de pertencimento a um grupo, cujos integrantes partilham interesses e atividades sociais”.	Clareza	Pertinência	Relevância
37	Durante as consultas de rotina, exames e sessões de quimioterapia, tem a presença de pessoas que lhe apoiam.	1,00	0,88	0,86
38	Se sente encorajada a realizar a quimioterapia quando tem a presença de pessoas que lhe apoiam.	0,94	0,96	0,94
39	A presença de pessoas da sua confiança durante a quimioterapia ajuda a diminuir a sensação de náusea, vômito, dores e outros sintomas.	0,98	0,98	0,96
40	Sempre tem profissionais disponíveis para lhe ajudar nas dúvidas e necessidades durante a quimioterapia.	0,96	0,94	0,90
41	Nos momentos de contato com os profissionais de saúde, sente que tem liberdade e abertura para expressar seus sentimentos e sensações sobre o tratamento e seu estado de saúde.	0,94	1,00	0,94
42	Se sente acolhida pelos profissionais quando realiza as sessões de quimioterapia.	1,00	0,94	0,94
43	Durante as sessões de quimioterapia, fez novos amigos que lhe apoiam durante o tratamento.	0,98	1,00	0,96
44	É ouvida quando tem dúvidas, angústias e preocupações sobre o seu estado de saúde e tratamento.	0,94	0,96	0,94
45	Recebe cuidados de pessoas próximas quando não consegue se cuidar sozinha.	0,98	0,98	0,96
46	Participa de atividades em grupo que lhe ajudam a se sentir mais confiante durante a quimioterapia.	0,98	0,96	0,94
47	Se sente segura por ter a presença de pessoas que cuidam de você durante as sessões de quimioterapia.	0,96	0,96	0,94
48	Se sente motivada a participar de atividades sociais com pessoas próximas a você.	0,98	0,98	0,92
Nº	**DIMENSÃO 5 - AUTOAPOIO** **(*Self-support*)** “Motivação pessoal para tomada de decisões, intenções intimas e pessoais relacionadas as situações vivenciadas”.	Clareza	Pertinência	Relevância
49	Se sente motivada a realizar as sessões de quimioterapia.	1,00	0,96	0,96
50	Se sente segura para tomar as decisões sobre seu tratamento.	1,00	0,98	0,96
51	Sente que seu humor e motivação para as atividades de lazer e convívio social se mantêm equilibrados durante a quimioterapia.	0,98	0,94	0,94
Nº	**DIMENSÃO 5 - AUTOAPOIO** **(*Self-support*)** “Motivação pessoal para tomada de decisões, intenções intimas e pessoais relacionadas as situações vivenciadas”.	Clareza	Pertinência	Relevância
52	Busca informações sobre a quimioterapia como forma de entender melhor como esta acontece e se sentir mais encorajada.	0,98	0,98	0,96
53	Tem pensamentos, atitudes e expectativas positivas sobre realizar o tratamento com quimioterapia.	0,98	0,98	0,96
54	Busca motivação na sua família, amigos e religião para superar os sentimentos de tristeza, decepção e dor.	1,00	0,98	0,96
55	Os efeitos adversos da quimioterapia são passageiros, e pensar sobre isso lhe faz sentir mais animada para finalizar o tratamento.	0,94	0,94	0,84
56	Compreende que as mudanças que acontecem no seu corpo durante a quimioterapia não são permanentes e não afetam sua autoestima.	0,96	0,98	0,88
57	Melhorou seu entendimento sobre a quimioterapia após passar pelo tratamento.	0,98	0,98	0,90
58	Se sente motivada a cuidar do meu corpo, aparência física e autoestima.	1,00	0,96	0,94
59	Busca na sua prática religiosa/espiritual motivação para enfrentar as sessões de quimioterapia.	1,00	0,98	0,96
60	Procura manter contato com amigos, vizinhos e família para apoio no enfrentamento da quimioterapia.	1,00	1,00	0,98

*CVC - Coeficiente de Validade de Conteúdo.*

Os resultados do CCI foram semelhantes ao estimado pelo CVC total, com concordância de 0,918 para o critério de clareza de conteúdo do instrumento, 0,957, para o critério de pertinência prática, e 0,967, para o critério de relevância teórica, em que todos os coeficientes foram estatisticamente significantes. A interpretação do CCI está relacionada à concordância das pontuações dos juízes, sendo o CCI igual a zero, quando não há concordância nenhuma na pontuação entre os avaliadores, e igual a um, quando há total concordância na pontuação entre os avaliadores. Observou-se que, em todos os itens, houve uma quase perfeita concordância (entre 0,8 e 1,00) em todos os domínios avaliados no instrumento, conforme a Tabela 1.

**Tabela 1:** Coeficiente de Validade de Conteúdo total e Coeficiente de Correlação Intraclasse segundo os domínios nas dimensões de clareza de linguagem, pertinência prática e relevância teórica, Recife, Pernambuco, Brasil, 2023

Domínios	Média (IC 95%)^*^	Valor de *p*
**Coeficiente de Validade de Conteúdo total**		
Clareza de conteúdo	0,944 (0,928 - 0,959)^a^	-
Pertinência prática	0,959 (0,949 - 0,969)^a^	-
Relevância teórica	0,931 (0,920 -0,941)^b^	-
**Coeficiente de Correlação Intraclasse**		
Clareza de conteúdo	0,918 (0,825 - 0,975)^a^	<0,001
Pertinência prática	0,957 (0,909 - 0,987)^a^	<0,001
Relevância teórica	0,967 (0,930 - 0,990)^a^	<0,001

* *Para a diferença entre os domínios, para letras iguais, não houve diferença significativa (p>0,05); IC 95% - Intervalo de Confiança de 95%.*

Com base na análise estatística e nas considerações dos juízes quanto à escrita e elaboração dos itens do instrumento, foram excluídos: os itens 05 e 21 (CVC <0,80 em pelo menos um dos critérios analisados); os itens 06, 09, 14, 28, 29 e 40 (a prática de apoio já estava contemplada em outro item do instrumento); o item 17 (não expressava ajuda concreta condizente a dimensão do apoio instrumental); e os itens 10, 18, 19 e 46 (prática de apoio atrelada a uma condição específica de vida, em que mulheres em tratamento quimioterápico podiam não vivenciar). Os itens 08, 15 e 16 apresentaram CVC <0,80 apenas no critério clareza. No segundo momento da TGN, por serem itens pertinentes e relevantes (CVC>80) para o instrumento, os juízes os mantiveram com a melhoria de sua escrita (Quadro 1). Após as eliminações, a segunda versão do instrumento contou com 47 itens, que foram submetidos à avaliação semântica junto ao público-alvo.

As oito mulheres participantes da avaliação semântica tinham entre 37 e 61 anos de idade, com média de 48 anos de idade, e todas afirmaram ser casadas. No tocante aos anos de estudo, observou-se que a formação escolar dessas mulheres estava compreendida desde o ensino fundamental incompleto até o ensino superior concluído.

Nessa avaliação, o IC médio foi de 0,99 pontos. Algumas participantes tiveram dificuldades na compreensão dos itens 16, 19, 35 e 46. Contudo, esses itens não foram excluídos, pois os autores optaram em mantê-los pela adequabilidade na avaliação do apoio ofertado pela rede social das mulheres com câncer de mama e pelos valores satisfatórios de ICs obtidos. Porém, ressalta-se que as sugestões foram acatadas, de modo que a redação desses itens foi ajustada para a clareza desses itens.

Mediante o término dessas etapas, configurou-se a terceira versão do instrumento de avaliação do apoio social às mulheres com câncer de mama em tratamento quimioterápico. Esta versão final (Apêndice) é composta por 47 itens, distribuídos entre as seguintes dimensões de apoio: emocional (oito itens); instrumental (sete); informativo (10); emocional (10); e autoapoio (12 itens).

## DISCUSSÃO

Os itens do instrumento foram desenvolvidos por meio de construto teórico relacionado às práticas de apoio da rede social às mulheres com câncer de mama em tratamento quimioterápico, que retratam as características do apoio presentes na literatura, especialmente em publicações provenientes da Ásia (Tailândia, Coreia do Sul e Japão), da Europa (Suécia) e da América (Brasil e Estados Unidos)^(16-24)^. Nos registros, os autores destacaram as práticas da rede social que podem oportunizar melhoria do bem-estar e qualidade de vida da mulher ao longo do tratamento^(14,16-24)^.

Com a etapa de revisão, que abrangeu estudos de vários continentes, foi possível estabelecer o conceito do objeto do estudo (práticas de apoio da rede social às mulheres com câncer de mama na quimioterapia), seus atributos constitutivos e operacionais, que nortearam o desenvolvimento dos itens do instrumento para, ao término da construção, operacionalizar o construto em itens^(25)^.

A construção e validade de um instrumento voltado à avaliação do apoio da rede social se configuram como um avanço significativo no cuidado em saúde, especialmente em oncologia. No contexto de assistir a mulher com câncer de mama em quimioterapia, incrementar o fazer profissional com ações não procedimentais permite que o cuidado seja integral e associado às particularidades de cada pessoa^(26)^.

As etapas de validação realizadas, a partir das recomendações dos juízes e do público-alvo, produziram um instrumento adequado e confiável para ser utilizado pelos profissionais de saúde e pela comunidade científica na melhoria da prática clínica e na investigação do apoio da rede social ofertado às mulheres com câncer de mama em quimioterapia. Apesar dessa importância, foram encontrados obstáculos para efetivação da participação dos juízes. Essa dificuldade de perda de juízes é uma das preocupações em estudos de validação, que são metódicos e demandam tempo significativo de contribuição^(10)^.

Na etapa de validade de conteúdo, observou-se que a titulação dos juízes se apresentou com maior ênfase no doutorado, com experiência acadêmica e assistencial, que conferiu maior confiabilidade nesta fase^(11,12)^. O uso da TGN favoreceu a adequação do instrumento por gerar resultados tangíveis dentro de um espaço relativamente curto. Porém, observou-se que, mesmo sendo viável para utilização no processo de validade de conteúdo, há necessidade de programação e execução em espaço de tempo curto, para que as particularidades do instrumento não sejam diluídas e, consequentemente, isso não resulte em perdas no consenso entre os juízes^(11,12)^.

As contribuições dos juízes na configuração dos itens foram pertinentes para organização das afirmativas nas dimensões de apoio estruturadas a partir do construto, especialmente na reformulação dos itens que apresentaram CVC relacionado à clareza inferior ao ponto de corte estabelecido no método (itens 08, 15 e 16). A sugestão para eliminação dos itens 5, 6, 9, 10, 14, 28, 29 e 40 foi acatada em virtude de que a essência do conteúdo desses itens já tinha sido contemplada em outras afirmações. O item 21 foi eliminado, pois o resultado do CVC foi insatisfatório. As eliminações de itens ocorreram também em outros estudos^(25,27)^, uma vez que havia similaridade da elaboração dos itens em diferentes dimensões, devido ao aprofundamento teórico do construto e aos coeficientes analisados não serem significativos.

O apoio dos colegas de trabalho é importante no processo de enfrentamento das mudanças oriundas do tratamento, fortalecimento do ideal de pertencimento a um grupo, autoestima e acolhimento^(5)^. Apesar disso, os itens 10, 18 e 19, que versavam sobre o apoio da rede primária relacionado ao desenvolvimento de atividades laborais durante a quimioterapia, foram excluídos porque, durante a indagação, a mulher participante do estudo poderia não ter condições de responder pelo fato de não trabalhar ou estar de licença de saúde.

As evidências comprovam que o afastamento do trabalho entre mulheres com câncer de mama é crescente, seja pelo Programa de Reabilitação Profissional da Previdência Social, pelo abandono do emprego devido ao agravamento do quadro de saúde ou pela vivência de preconceito e outras experiências negativas^(28)^.

O item 46, voltado à realização de atividades em grupo pela mulher durante a quimioterapia, teve eliminação sugerida pelos juízes, uma vez que ela pode não ter indicação para participar dessas atividades. O desenvolvimento da quimioterapia ocasiona toxicidade hematológica, que culmina em prejuízos na vida da mulher, que pode apresentar redução da imunidade em virtude da diminuição dos elementos figurados do sangue e oportunizar o aparecimento de leucopenia, trombocitopenia, neutropenia febril e anemia^(29)^. Portanto, as atividades sociais em grupo e coletividade devem ser restritas na ocorrência desses quadros, por oportunizarem risco de comorbidades ao longo do tratamento^(30)^.

A avaliação semântica oportunizou modificações relacionadas à redação de algumas palavras/termos nos itens 16, 19, 35 e 46, com vistas a uma melhor compreensão das questões colocadas pelo público-alvo a que o instrumento se destina. As sugestões foram acatadas e incorporadas na versão final. Esta etapa pode ser compreendida como um avanço importante para translação do conhecimento de enfermagem, especialmente em estudos que desenvolvem tecnologias para o cuidado. A literatura destaca uma preocupação cada vez maior em realizar pesquisas com pacientes em contextos reais, com o objetivo de aumentar os sistemas de linguagem padronizada e impulsionar sua translação para prática^(31)^.

Os critérios adotados para validade de conteúdo e avaliação semântica neste estudo se basearam em referências que permitiram uma avaliação criteriosa da fidedignidade dos itens e na produção de um instrumento confiável e adequado para utilização no cuidado da mulher com câncer de mama. O processo percorrido para validação de um instrumento é determinante na aplicação da medida para qual este se propõe^(10)^. É fundamental a realização de etapas de validação para determinar a legitimidade e credibilidade dos resultados de um estudo e o reconhecimento da qualidade dos itens de um instrumento^(32)^.

### Limitações do estudo

Apesar de atender ao tamanho amostral preconizado pela literatura^(10)^, considerou-se como limitação do estudo a quantidade de juízes que colaboram com o segundo momento da TGN, uma vez que um maior número de juízes possibilitaria uma discussão mais apurada e exaustiva do instrumento, de forma a aperfeiçoar, ainda mais, o produto construído neste estudo.

### Contribuições para a área da saúde, enfermagem ou políticas públicas

A utilização deste instrumento na prática clínica do enfermeiro voltada ao cuidado da mulher em tratamento quimioterápico fornece parâmetros na avaliação do suporte da rede social dessa mulher. Essa avaliação pode subsidiar ações de mobilização da rede social, primária e secundária, de modo a implementar intervenções necessárias para que os apoios ofertados pela rede social da mulher com câncer mamário se convertam em benefícios reais no enfrentamento desta neoplasia. Além disso, este instrumento validado pode ser aplicado em estratégias de ensino nos cursos de graduação em enfermagem, como também pode ser usado nas pesquisas científicas sobre mulheres com câncer de mama em tratamento quimioterápico.

## CONCLUSÕES

O instrumento disponibilizado neste estudo aos profissionais de oncologia é capaz de avaliar o apoio da rede social às mulheres com câncer de mama durante o seguimento da quimioterapia. O seu processo de construção e validação, baseado em referenciais teórico e metodológico, conceituados pela literatura, evidencia que essa ferramenta de trabalho foi validada, é segura e adequada para que se destina.

A definição da estrutura conceitual permitiu a identificação das práticas de apoio da rede social, de modo a aproximá-las das particularidades vivenciadas pela mulher com câncer de mama durante a quimioterapia na construção dos itens do instrumento. A identificação de evidências de validade de conteúdo garantiu a adequabilidade dos itens quanto à clareza, pertinência e relevância, tornando o instrumento confiável na investigação do fenômeno do apoio da rede social, de forma segura, pelos profissionais na sua rotina assistencial. A avaliação semântica, por sua vez, conferiu clareza redacional para compreensão dos itens do instrumento pelo público-alvo. Porém, recomenda-se a realização de estudo de validação clínica do referido instrumento.

## Data Availability

Os dados de pesquisa estão disponíveis no corpo do artigo.
